# Association between sickle cell and β-thalassemia genes and hemoglobin concentration and anemia in children and non-pregnant women in Sierra Leone: ancillary analysis of data from Sierra Leone’s 2013 National Micronutrient Survey

**DOI:** 10.1186/s13104-018-3143-x

**Published:** 2018-01-17

**Authors:** James P. Wirth, Rashid Ansumana, Bradley A. Woodruff, Aminata S. Koroma, Mary H. Hodges

**Affiliations:** 1 GroundWork, Fläsch, Switzerland; 2 Mercy Hospital Research Laboratory, Kulanda Town, Bo, Sierra Leone; 3 Ministry of Health and Sanitation, Freetown, Sierra Leone; 4 Helen Keller International, Freetown, Sierra Leone

**Keywords:** Sickle cell, β-thalassemia, Hemoglobin, Anemia, Children, Non-pregnant women, Sierra Leone

## Abstract

**Objective:**

By measuring the associations between the presence of sickle cell and β-thalassemia genes, we assessed the extent to which these hemoglobinopathies contribute to the high prevalence of anemia observed in preschool-aged children and women of reproductive age in Sierra Leone.

**Results:**

The prevalence of anemia was statistically significantly higher in children with homozygous sickle cell genes (HbSS) than in children with normal hemoglobin genes (HbAA or HbAC), but there was no difference in anemia prevalence in those with heterozygous sickle cell trait (HbAS or HbSC) compared with those with normal hemoglobin genes. In women, there was no difference in anemia prevalence by sickle cell status. In both children and women, there was no difference in the anemia prevalence for individuals with or without the β-thalassemia gene. For both sickle cell and β-thalassemia, there was no significant difference in hemoglobin concentrations by sickle cell or β-thalassemia status. Anemia prevalence was higher in children and women with homozygous sickle cell (HbSS). However, as the prevalence of HbSS children (5.4%) and women (1.6%) was quite small, it is unlikely that these hemoglobinopathies substantially contributed to the high anemia prevalence found in the 2013 national micronutrient survey.

## Introduction

Sierra Leone’s Micronutrient Survey (SLMS) was conducted in late 2013. A key objective of the SLMS was to identify factors associated with anemia. The anemia prevalence rates in children (hemoglobin < 110 g/L) of 76.3% and women (hemoglobin < 120 g/L) of 44.8% indicate a severe public health problem according to criteria developed by the World Health Organization (WHO) [1]. The SLMS also found that the prevalence of iron deficiency, a supposed primary cause of anemia, was relatively low in children (5.2%) and non-pregnant women (8.3%) [2]. The prevalence of iron deficiency anemia was accordingly low, at 3.8% in children and 6.1% in women [2].

Malaria and inflammation were associated with anemia in both children and women, while iron deficiency was associated with anemia in women only [3]. These associations, however, only explained one-quarter of the anemia observed. Hemoglobinopathies were identified as plausible contributors to anemia, as a previous study in Sierra Leone found that 22% of participants were found to have either homozygous or heterozygous sickle cell [4]. Moreover, an analysis conducted by WHO estimates that, in Sierra Leone, more than 19 infants per 1000 births are born with a “major haemoglobinopathy” [5].

There are various types of hemoglobinopathies, and the most widespread occur from inherited sickle cell and/or thalassemia genes. Table 1 provides the various hemoglobinopathies examined by our study, nomenclature, and commonly described sequelae. Our post hoc analysis of the associations between hemoglobinopathies and hemoglobin concentrations and anemia prevalence aims to assess how much hemoglobinopathies contribute to the high prevalence of anemia observed in preschool-aged children and women of reproductive age in Sierra Leone.

**Table 1:** Categorization and sequelae of hemoglobinopathies examined

Category	Nomenclature	Definition; sequelae
Normal hemoglobin (AA) or hemoglobin C trait (AC)	HbAA/AC	Red blood cells are normal, and individuals have no symptoms and normal hemoglobin concentrations [9, 15]
Homozygous sickle cell genes	HbSS	Sickle cell gene inherited from both parents, resulting in red blood cells that have a sickle shape at low oxygen tension; HbSS can result in increased under-5 mortality and maternal mortality, and hemoglobin concentrations are often around 80 g/L [9, 16]
Heterozygous sickle cell trait	HbSC	Heterozygous sickle cell trait, whereby individuals inherit a sickle cell gene from one parent and a hemoglobin C gene from the other parent; individuals have fewer sickle cells but may have slightly lower hemoglobin concentrations than HbAA individuals [17]. Hemoglobin C is most prevalence in West Africa [15]
Heterozygous sickle cell trait	HbAS	Heterozygous sickle cell trait, whereby individuals inherit a normal hemoglobin A gene from one parent and a hemoglobin S gene (i.e. sickle cell gene) from the other parent; Individuals often no symptoms and normal hemoglobin concentrations [6] and inherit partial protection against malaria [18]
β-Thalassemia	β-Thalassemia	An inherited mutation that results in a “reduced or absent synthesis of beta globin chains” [19]; red blood cells are smaller than usual and hemoglobin may be lower than in HbAA individuals [20]

## Main text

### Blood collection, storage, and laboratory analyses

Hemoglobin concentration was measured on-site using a portable hemoglobinometer (HB201+, Hemocue AB, Ängelholm, Sweden). In children, blood was collected directly from the heel or finger using a lancet stick. Following hemoglobin measurement, approximately 300 µL of capillary blood was collected in a potassium-EDTA-coated microtube (Microvette CB 300, Sarstedt, Switzerland). In non-pregnant women, hemoglobin was measured using a few drops of blood from the 5 mL of blood collected by venipuncture into an potassium-EDTA-coated tube (Vacutainer^®^, Becton–Dickinson, Germany). Blood was expressed from the tube into a weighing boat using a DIFF-Safe™ blood dispenser.

Following centrifugation, blood pellets were stored at – 20 °C and remained frozen for approximately 3 years until analysis. Although blood pellet samples were initially available for the 654 children and 774 women who provided blood samples during the SLMS [3], some samples had insufficient quantity for analysis, were poorly labeled, or had been inadvertently discarded or misplaced during the Ebola outbreak due to increased need for freezer space and prioritization of Ebola-related laboratory testing. As a result, this present study was conducted in a non-random sub-sample of 388 children and 255 women for whom samples with sufficient volume were still available. All available samples were tested without regard to the results of testing for inflammation or malaria.

Hemoglobinopathy analyses were done at the Mercy Hospital research laboratory. DNA extractions were done from the blood pellets using Masterpure™ DNA purification kit for blood (Epicentre, 5602 Research Park Blvd., Suite 200, Madison, WI 53719 U.S.A.). Extracted DNA was measured with Qubit and Nanodrop to ensure that they measured 50 ng/µL or more. β-Globin Strip Assay IME^®^ (ViennaLab Diagnostics GmbH, Vienna Austria) was used for initial detection of β-thalassemia. Himedia diagnostics for sickle cell and β-thalassemia (HiMedia Laboratories Pvt. Limited, Mumbai-400 086, India) were also used. Results for the Himedia diagnostics for both sickle cell analyses and β-thalassemia assays were obtained by agarose gel electrophoresis using prepared gel or Flashgel.

The SLMS survey protocol, was approved by the Sierra Leone Ethics and Scientific Review Committee of Sierra Leone’s Ministry of Health and Sanitation, and written informed consent was required of women and the caretakers of children to participate in the SLMS. Details of the design and data collection procedures of the SLMS are described elsewhere [2, 3].

### Case definitions and statistical analysis

In children, any anemia was defined as hemoglobin concentration < 110 g/L. Mild anemia was defined as 100–109 g/L, moderate anemia as 70–90 g/L, and severe anemia as < 70 g/L. In women, any anemia was defined as hemoglobin concentration < 120 g/L. Mild anemia was defined as 110–119 g/L, moderate anemia as 80–109 g/L, and severe anemia as < 80 g/L.

The statistical precision of prevalence estimates was assessed using 95% confidence intervals. The statistical significance of differences in means between subgroups was assessed using ANOVA, and that of differences in the prevalence of anemia were assessed using Chi square. All analyses were unweighted; however, all calculation of measures of precision, including p values and confidence intervals, accounted for the complex sampling employed in the SLMS. Data analysis was done using SPSS version 24 with the complex survey module.

## Results

### Children

Results of sickle cell and β-thalassemia testing, along with the individual and household data required to calculate measures of precision, were available for 388 (59%) of the total sample of 654 children included in the SLMS who had contributed samples with sufficient volume for analysis of hemoglobinopathies. The mean hemoglobin in these 388 children was 99.0 g/L (95% CI 96.7, 101.4), and 74.0% (95% CI 67.7, 79.4) of children were anemic. Severe anemia was relatively rare, affecting only 2.3% (95% CI 1.1, 5.0) of these 388 children. Moderate and mild anemia were more widespread, affecting 44.8% (95% CI 38.4, 51.5) and 26.8% (95% CI 21.8, 32.5) children, respectively.

Overall, 21 (5.4%; 95% CI 3.7, 8.0) of 388 children tested for hemoglobinopathies had HbSS, 61 (15.7%; 95% CI 12.6, 19.5) had heterozygous sickle cell trait, and 306 (78.9%; 95% CI 74.8, 82.4) had either HbAA or HbAC. β-thalassemia was found in 64 (16.5%; 95% CI 12.7, 21.2) of 388 children. Concurrent sickle cell and β-thalassemia genes were found in 11 (2.8%) children, of whom only one had HbSS. The mean hemoglobin concentrations in children with HbSS, heterozygous sickle cell trait, and normal hemoglobin are not statistically significantly different (see Table 2). In addition, no statistically significant difference in anemia prevalence was found between children with heterozygous sickle cell trait and those with either HbAA or HbAC. However, the anemia prevalence in HbSS children was statistically significantly higher than in children with normal hemoglobin (i.e. HbAA or HbAC), as indicated the risk ratio confidence intervals do not touch 1. There was minimal difference in the mean hemoglobin concentration and the prevalence of anemia in children with versus without the β-thalassemia gene.

**Table 2:** Mean hemoglobin and prevalence of anemia in children 6–59 months of age, by presence of sickle cell and β-thalassemia mutations, Sierra Leone, 2013

	N^a^	Mean Hb (g/L)	p value	% Anemia	Risk ratio	Risk ratio (95% CI)
Sickle cell						
HbAA or HbAC	306	99.0	0.559	73.5	Reference	–
HbAS or HbSC	61	99.9		70.5	0.96	(0.81, 1.1)
HbSS	21	96.5		90.5	1.2	(1.05, 1.4)
β-Thalassemia						
No	324	98.7	0.280	74.1	Reference	–
Yes	64	100.9		73.4	0.99	(0.84, 1.2)

^a^ The n’s are un-weighted denominators for each subgroup

### Women

Results of sickle cell and β-thalassemia testing, along with the individual and household data required to account for the complex sampling, were available for 255 (32.8%) of the total sample of 776 non-pregnant women in the SLMS who provided a venous blood sample with sufficient volume for analysis of hemoglobinopathies. The mean hemoglobin in these 255 women was 122.2 g/L (95% CI 120.0, 124.3), and 40.8% (95% CI 33.2, 48.89.7) of women were anemic. As with children, severe anemia was rare; only one woman (0.4%; 95%CI 0.1, 2.9) had severe anemia. Moderate anemia was found in 16.5% (95% CI 11.4, 23.3) of women. Mild anemia was found in 23.9% (95% CI 17.6, 31.7) of women, which accounts for more than half of all anemia in women.

Only 4 women (1.6%; 95% CI 0.6, 4.1) had HbSS, and 20 (7.8%; 95% CI 5.0, 12.2) had heterozygous sickle cell trait. The 231 remaining women (90.6%; 95% CI 86.8, 93.4) had either HbAA or HbAC. β-thalassemia was found in 24 women (9.4%; 95% CI 5.3, 16.2), and concurrent sickle cell and β-thalassemia genes were observed in two women (0.8%). The mean hemoglobin concentrations among women with heterozygous sickle cell trait or HbSS were 2.1 and 4.1 g/L, respectively, lower than concentrations in women with HbAA or HbAC; however, these differences were not statistically significant (Table 3). In addition, there was no statistical significance in the differences in mean hemoglobin concentrations between women with β-thalassemia and those without this mutation. Women with HbSS had almost twice the risk of being anemic than women with HbAA or HbAC; however, this difference was also not statistically significant. The prevalence of anemia in women without β-thalassemia was higher than in women with β-thalassemia, but with no statistical significance.

**Table 3:** Mean hemoglobin and prevalence of in non-pregnant women 15–49 years of age, by presence of sickle cell and β-thalassemia mutations Sierra Leone, 2013

	n^a^	Mean Hb (g/L)	p value	% Anemia	Risk ratio	Risk ratio (95% CI)
Sickle cell						
HbAA or HbAC	231	122.4	0.598	39.4	Reference	–
HbAS or HbSC	20	120.3		50.0	1.3	(0.81, 2.0)
HbSS	4	118.5		75.0	1.9	(0.98, 3.7)
β-Thalassemia						
No	231	122.3	0.768	41.6	Reference	–
Yes	24	121.3		33.3	0.80	(0.44, 1.5)

^a^ The n’s are un-weighted denominators for each subgroup

## Discussion

Our analysis found that the only difference in children or women which had statistical significance was in the prevalence of anemia in children with sickle cell disease (HbSS) compared to children with normal hemoglobin. However, the difference in anemia prevalence between women with HbSS and women with normal hemoglobin was substantial and almost statistically significant. Although anemia prevalence is higher in children and women with HbSS, sickle cell disease was found in relatively few children and women. As such, the contribution of sickle cell disease to the overall prevalence of anemia observed in the SLMS is low.

In both children and women, there were only minor, non-statistically significant differences in hemoglobin concentration and anemia prevalence in individuals with or without the β-thalassemia gene. As a result, β-thalassemia also probably plays little role in contributing to anemia in the SLMS subjects.

The combined prevalence of sickle cell disease and trait in women (9.4%) in our study is comparable to the 10–15% estimate presented by Wellems & Fairhurst [6], who mapped the frequency of the HbS allele for the African continent. The combined prevalence in children (21.1%), however, is markedly higher than Wellems & Fairhurst’s estimate and other studies from the region. To illustrate, a hospital-based study in Monrovia found that 11.5% of newborns had either sickle cell disease or trait (HbSS = 1.2%; HbAS = 10.3%) [7], and a population-based study in children in Guinea-Bissau found only 4.9% had sickle cell disease or trait (HbSS = 0.2%; HbAS = 4.7%) [8]. Although our findings could suggest that Sierra Leone has a higher prevalence of the HbS allele than its neighboring countries, our results are not truly representative of the population because of the convenience selection of blood pellet samples for testing.

The prevalence of sickle cell disease (HbSS) is also higher among children that adult women. While part of this difference is undoubtedly due to increased mortality stemming from sickle cell disease in childhood [9, 10], the prevalence in children is still notably higher than the prevalence found in other studies, as discussed above.

Similar to sickle cell, the prevalence of β-thalassemia among children and women in our study was notably higher that the prevalence found in other studies. Mockenhaupt et al. [11] found heterozygous β-thalassemia in 1% of pregnant women enrolled in a cross-sectional study in Ghana Two studies from the 1970’s in Nigeria found a β-thalassemia prevalence of < 1% [12]; however, in a small clinic-based study in Nigeria, Vincent et al. [13] found that 6% of adults possessed both sickle cell and β-thalassemia genes. A similar prevalence of β-thalassemia has been observed in Pakistan (5–8%, [14]) and Indonesia (6%, [13]).

## Limitations

Despite the relatively high prevalences observed, our study indicates that the genetic hemoglobinopathies of sickle cell and β-thalassemia may not make substantial contributions to anemia in Sierra Leone. However, our sample size is small and many samples collected as part of a representative population-based survey were lost or were inadequate due to hemolysis as well as degradation during prolonged storage. As a result, our results do not properly represent the population of Sierra Leone. In addition, hemoglobinopathy testing was restricted to only two of several possible abnormalities of hemoglobin. Most importantly, alpha thalassemia was not measured.

In spite of these limitations, this study demonstrates that hemoglobinopathies do not account for a high proportion of anemia in children or women. In addition, this study builds upon the results of the SLMS, which found malaria and inflammation only explained about 25% of the population attributable risk of anemia in children and women, and that micronutrient deficiencies did not contribute to anemia [3]. Other causes of anemia, which may be amenable to program intervention, should be investigated in order to identify the most effective way to decrease the prevalence of anemia in children and women in Sierra Leone.

## Abbreviations

ANOVA: analyses of variance
CI: confidence interval
DNA: deoxyribonucleic acid
EDTA: ethylenediaminetetraacetic acid
g/L: gram per liter
HBAA: normal hemoglobin genes
HBAC: normal hemoglobin genes
HBSS: sickle cell genes
HBSC: heterozygous sickle cell genes
SLMS: Sierra Leone Micronutrient Survey
WHO: World Health Organization

## References

[1] World Health Organization. Haemoglobin concentrations for the diagnosis of anaemia and assessment of severity. Vitamin and mineral nutrition information system (WHO/NMH/NHD/MNM/11.1). 2011. http://www.who.int/vmnis/indicators/haemoglobin.pdf. Accessed 30 Jan.

[2] MOHS, UNICEF, Helen Keller International, GroundWork, WHO. 2013 Sierra Leone Micronutrient Survey. Freetown; 2015.

[3] Wirth JP, Rohner F, Woodruff BA, Chiwile F, Yankson H, Koroma AS (2016). Anemia, micronutrient deficiencies, and malaria in children and women in Sierra Leone prior to the ebola outbreak—findings of a cross-sectional study. PLoS ONE.

[4] Wurie AT, Wurie IM, Gevao SM, Robbin-Coker DJ (1996). The prevalence of sickle cell trait in Sierra Leone. A laboratory profile. West Afr J Med..

[5] WHO. Genes and human disease. Genomic resource centre. 2017. http://www.who.int/genomics/public/geneticdiseases/en/index2.html. Accessed 31 May 2017.

[6] Wellems TE, Fairhurst RM (2005). Malaria-protective traits at odds in Africa?. Nat Genet.

[7] Tubman VN, Marshall R, Jallah W, Guo D, Ma C, Ohene-Frempong K (2016). Newborn screening for sickle cell disease in Liberia: a pilot study. Pediatr Blood Cancer.

[8] Masmas TN, Garly ML, Lisse IM, Rodriques A, Petersen PT, Birgens H (2006). Inherited hemoglobin disorders in Guinea-Bissau, West Africa: a population study. Hemoglobin..

[9] WHO (2006). Sickle-cell anaemia.

[10] Weatherall D, Akinyanju O, Fucharoen S, Olivieri N, Musgrove P, Jamison DT (2006). Chapter 34. Disease control priorities in developing countries.

[11] Mockenhaupt FP, Rong B, Günther M, Beck S, Till H, Kohne E (2000). Anaemia in pregnant Ghanaian women: importance of malaria, iron deficiency, and haemoglobinopathies. Trans R Soc Trop Med Hyg..

[12] Akinyanju OO (1989). A profile of sickle cell disease in Nigeria. Ann N Y Acad Sci.

[13] Susanti AI, Sahiratmadja E, Winarno G, Sugianli AK, Susanto H, Panigoro R (2017). Low hemoglobin among pregnant women in midwives practice of primary health care, Jatinangor, Indonesia: iron deficiency anemia or β-thalassemia trait?. Anemia..

[14] Muhammad R, Shakeel M, Rehman SU, Lodhi MA (2017). Population-based genetic study of β-thalassemia mutations in Mardan Division, Khyber Pakhtunkhwa Province, Pakistan. Hemoglobin..

[15] Piel FB, Howes RE, Patil AP, Nyangiri OA, Gething PW, Bhatt S (2013). The distribution of haemoglobin C and its prevalence in newborns in Africa. Sci Rep..

[16] Asnani MR, McCaw-Binns AM, Reid ME (2011). Excess risk of maternal death from sickle cell disease in Jamaica: 1998–2007. PLoS One..

[17] Lepira FB, Mukendi TK, Mbutiwi FIN, Makulo JR, Sumaili EK, Kayembe PK (2016). Sickle cell trait, hemoglobin levels and anemia among black patients with predialysis chronic kidney disease: a post hoc analysis. World J Cardiovasc Dis..

[18] Luzzatto L (2012). Sickle cell anaemia and malaria. Mediterr J Hematol Infect Dis..

[19] Cao A, Galanello R (2010). Beta-thalassemia. Genet Med..

[20] Muncie HL, Campbell J (2009). Alpha and beta thalassemia. Am Fam Physician.

